# Draft Genome Sequence of the Dimorphic Prosthecate Bacterium *Brevundimonas abyssalis* TAR-001^T^

**DOI:** 10.1128/genomeA.00826-13

**Published:** 2013-10-17

**Authors:** Taishi Tsubouchi, Shinro Nishi, Keiko Usui, Yasuhiro Shimane, Yoshihiro Takaki, Tadashi Maruyama, Yuji Hatada

**Affiliations:** Japan Agency for Marine-Earth Science and Technology, Yokosuka-shi, Kanagawa, Japan

## Abstract

We report the 3.0-Mb draft genome sequence of *Brevundimonas abyssalis* strain TAR-001^T^, isolated from deep-sea floor sediment. The draft genome sequence of strain TAR-001^T^ consists of 2,979,700 bp in 128 contigs, with a G+C content of 68.2%, 2,946 potential coding sequences (CDS), 3 rRNAs, and 41 tRNAs.

## GENOME ANNOUNCEMENT

Bacterial adhesion is the first station node of microbial colonization or the formation of biofilm (1), composed of cells and the extracellular polysaccharide (EPS) they secrete (2). The mechanism for mediating attachment to surfaces depends on the physiological features of other microbes. However, macromolecules, such as EPS or filamentous cell appendages (3), function to form a bridge between the microbial cell and the surface; therefore, they are essential factors for bacterial adhesion. In the aquatic bacterium *Caulobacter crescentus*, which is famous for its strong adherent properties (4), three extracellular appendages, flagellum, pili, and holdfast, are required, and their biosynthesis is regulated at the level of the cell cycle. The polysaccharide component of the holdfast is composed in part of oligomers of *N*-acetylglucosamine (5, 6). *Brevundimonas abyssalis* strain TAR-001^T^ (7), from the family *Caulobacteraceae*, possesses three polar structures, like *C. crescentus* strain CB15^T^, and these structures show adhesiveness to one another and to the surfaces of abiotic substances.

The draft genome sequencing of *B. abyssalis* TAR-001^T^ was performed on an Ion Torrent PGM sequencer (Life Technologies) equipped with a 318 chip. Data from the genomic DNA library contained 573,064 reads and 170,211,700 nucleotide bases, with an average read length of 297.02 bp, obtained using 400-base chemistry. Assembly using the CLC Genomics Workbench version 6.01 (CLC bio Japan, Inc.) generated 128 contigs, with maximum and minimum contig sizes of 135,343 bp and 535 bp, respectively.

The draft genome sequence comprising 2,979,700 nucleotides was annotated with the help of MetaGeneMark (8) and the Rapid Annotations using Subsystems Technology (RAST) server (9). These annotation results indicate that strain TAR-001^T^ possesses the gene coding for PodJ, which does not exist in *C. crescentus* CB15^T^ or *Brevundimonas diminuta* ATCC 11568^T^, in addition to a set of genes coding for a *Caulobacter*-type cell cycle, and all of these annotated genes show 24.2 to 91.0% identity. However, *rseP*, which codes for zinc metalloprotease, was not detected. The PodJ and zinc metalloprotease proteins are considered to contribute following the polar morphogenesis and pilus biosynthesis processes. As for flagellar assembly, strain TAR-001^T^ contains almost all of the necessary genes; however, *flgF*, *flgG*, and *flgH*, which code for the flagellar basal body rod proteins FlgF, FlgG, and FlgH, respectively, consisting of an outer membrane junction unit, were not found. Strain TAR-001^T^ has genes coding for the holdfast synthesis proteins: glycosyltransferase family protein, polysaccharide biosynthesis protein, polysaccharide deacetylase, and holdfast attachment proteins HfaA, HfaB, and HfaD. We could not find the genes coding for HfsA, HfsB, and HfsD, which are membrane periplasmic auxiliary protein 1, a tyrosine autokinase, and a membrane channel protein, respectively, and concert to export synthesized polysaccharide; however, *C. crescentus* CB15^T^ and *B. diminuta* ATCC 11568^T^ possess these genes.

### Nucleotide sequence accession numbers.

The draft genome sequence of *B. abyssalis* TAR-001^T^ reported in this paper has been deposited in DDBJ/EMBL/GenBank under the accession no. BATC00000000 (accession no. of contigs are BATC01000001 to BATC01000128) in BioProject no. PRJDB1125.
